# Surgery, Ophthalmology and Otology

**Published:** 1875-10

**Authors:** 


### II. Surgery, Ophthalmology and Otology.

1. *Plugging the Nasal Cavities.* J. English. (*Centralblatt*, f. Chir., No. 34, 1875.)

The writer enumerates all the external and internal remedies that have ever been used against nose-bleeding and shows how, after all, Bellocq's tube had always to be resorted to as the surest method of plugging the nasal cavity. But it is not unobjectionable, because of the annoying salivation produced by the thread which is carried out through the mouth. This objection is obviated by using a rubber bag attached to a flexible rubber tube ; introduced through the nostril into the naso-pharyngeal cavity, the rubber tampon is filled with water and then drawn up tightly into the posterior nares. A clamp attached to the rubber tube prevents the escape of the water.

Dr. T. H. Jewett describes (*Philadelphia Med. and Surg. Reporter*, Sept. 11), the following simple plug in nasal hæmorrhage: "Roll up a lock of cotton into a cylinder an inch or an inch and a half in length ; tie a strong thread to the middle of the roll ; bring the two ends of the roll together, and then opening the nasal orifice pass the middle or folded part of the roll into the nostril ; next, with the blunt end of a lead pencil press in the cotton roll slowly, along the floor of the nose, one inch or more, and rest. If the blood passes down into the throat, you may be sure the bleeding spot is behind the roll, so push in your roll further and the blood will cease to pass behind. Then, holding on to the string, pass some loose cotton into the nostril and push it down to the plug. The cotton will swell with the moisture and arrest the hæmorrhage. In a day or two the natural secretions of the nasal surfaces will loosen the plug and it may be easily removed by the string.

2. *Tannic Acid for Acute Conjunctivitis.* E. Emmert. (*Aug. Med. Central Zeitung*, No. 64, 1875.)

For severe conjunctivitis, in scrofulous children, with much swelling of the eyelids, intolerance of light and muco-watery secretion, a solution of tannic acid (ʒ ss to ℥ j) instilled into the eyes every two hours, acted more efficiently and arrested the inflammation more promptly than any other remedy. In cases of blennorrhœal and gonorrhœal conjunctivitis the tannic acid accomplished just as much as arg. nitr., and the doctor is in favor of tannic acid, because it can be given to the patients to be used at home, which we can never do with the arg. nitr.; in country practice, therefore, he thinks the tannic acid will be greatly used for the above diseases.

[Tannic acid was a favorite remedy at the County Hospital under Dr. Hildreth; he used a saturated solution of tannic acid in glycerine and bromide of ammonia. But a blennorrhœal eye requires the daily attendance of the physician, just as much as a case of typhoid fever, and then arg. nitr. can be used just as well.—Ed.]

3. *Transplantation of the Conjunctiva.* O. Becker. (*Wiener Med. Wochenschrift.*)

Prof. Otto Becker reports two cases of adhesion of the eyelid to the globe, in which, after failing with other operations, he obtained a satisfactory result from the above transplantations. He carefully dissected the lid from the globe and implanted into the defect of the ocular conjunctiva a piece of conjunctiva just removed from a white rabbit, and secured it in its new place by four sutures. For the sutures very fine and strongly curved needles and the finest untwisted white silk were used. In one case, the whole implanted piece healed in; in the second case, only about one-third of the flap became adherent, but still the result as to the mobility of the eye was satisfactory.

4. *Grave's Disease.* R. Bartholow. (*Chic. Jour. Nerv. Dis.*, July, 1875.)

Before the American Med. Association, Robert Bartholow, M.D., read an interesting paper on exophthalmic goitre. Sustaining the theory that this disease is a purely functional disorder of the sympathetic nerve, he gives the detailed history of three cases which were materially benefited by a regular course of treatment with the galvanic current. The negative pole was placed on the epigastrium, and the positive was so applied as to include the cervical sympathetic, the pneumogastric and the cilio-spinal region within the circuit. The *seances* were ten minutes in duration.

5. *Elastic Ligature for the cure of webbed fingers.* VOGEL. (*Am. Jour. Med. Sci*, July, 1875.)

Dr. M. Vogel tried this treatment with a great deal of success in a very bad case of webbed fingers. The first rubber thread was entered just above the last phalanx and tied over the tip of the united fingers, care being taken that it occupied an exactly intermediate position between them. After eight days the phalanges were completely separated. The necessary tightening of the ligature was done from time to time by placing a small roll of plaster under it. When the fingers were completely separated by this process they were fixed on a pasteboard splint, while, to prevent the re-formation of a web at the junction of the fingers, an elastic thread was attached to a wristband and gently stretched between them. Cicatrization went on rapidly, and after a few weeks the fingers had regained a natural appearance.

Another case of webbed fingers treated successfully by the elastic ligature, is reported by Prof. Dittell.—*Philad. Med. and Surg. Reporter.*

6. *Strangulated Hernia reduced by taxis through colon.* A. HADDEN. (*N. Y. Med. Record*, July 24.)

Alex. Hadden (New York) relates a case of strangulated direct inguinal hernia which, after failing with other methods, he reduced by taxis through the colon in this way :

"The patient being fully under the influence of chloroform, was placed on her chest and knees, and supported in that position; I next introduced my fingers into the anus, and passed my hand by gentle pressure up into the colon. I had some difficulty in following the intestine over the promontory of the sacrum, but when my hand had passed this point it was very free. I could feel the engorged intestine plainly, and could make traction on it with my fingers, and did carefully, fearing that I might rend it. My manipulations consisted, chiefly, in gently rubbing my finger along the intestine from the inner surface of the ring, and using external taxis with my other hand at the same time. The desired result was accomplished in about ten or fifteen minutes with comparative ease."

7. *Treatment of Strangulated Hernia by Subcutaneous Dilation.* H. R. Allen. (*Philad. Med. and Surg. Reporter*, July 10, 1875.)

This practice was advised by Seutin in 1856, and again by M. Langenbeck in 1864, but it never could gain any prominence in surgery. Now Dr. H. R. Allen (San-Francisco, Cal.,) reports a few cases of severe strangulation which he and other physicians failed to relieve by the ordinary taxis, and which he then speedily released by introducing his index finger forcibly into the ring and distending it subcutaneously. Upon his experience in these cases he devised an instrument for the forcible dilatation or subcutaneous laceration of the stricture. For a full description of this instrument, see *Reporter*.

8. *Simon's Method of Dilating the Female Urethra.* P. Bruns. (*Centralblatt, d. Chir.* 33.)

Prof. Simon (Heidelberg) published, a short time ago, a method of quickly and safely dilating the female urethra for the purpose of examining the bladder with specula as well as the finger. In the first place the external orifice of the urethra is enlarged by two lateral incisions (one-eighth of an inch deep); then the urethra itself is dilated by the introduction of a series of seven conical specula of hard rubber, the No. 1 has a diameter of one-quarter of an inch, and No. 7 measures one and one-quarter inches. Directly after withdrawing the largest speculum the index finger is introduced for palpating the interior of the bladder. According to S. even the narrowest urethra can thus be dilated within a few minutes; he has made use of his method in over sixty cases, and an incontinence of the bladder has not been experienced in a single one.

To illustrate the great usefulness of Simon's method, Dr. Bruns gives the following case: A girl, 24 years of age, had a hair pin in her bladder, and three unsuccessful attempts at extraction had been made. In the narcosis the external orifice of the urethra was enlarged by two superficial cuts, and the seven specula were introduced successively. The index finger then followed, and with it a thin forceps could be introduced into the bladder. Under the guide of the finger the hair pin was caught up without difficulty and extracted. The whole operation occupied five minutes. No incontinence of urine after the operation.

9. *The Aspirator in Cases of Retention of Urine.* P. I. CONNER. (*Cincinnati Clinic*, July 10.)

P. I. Conner read a paper on this subject before the Ohio State Medical Society. Whenever by a tight stricture, hypertrophy of the prostate, or other causes, the distended bladder cannot be emptied by catheterization, he recommends the tapping of the bladder through the supra-pubic space by the needle of the aspirator. The method is certain, easy, quick and safe, and attended by very little pain. He has tapped and emptied the bladder by aspiration as often as ten times within two weeks without any unpleasant consequences.

10. *Extirpation of a Tumor of the Bladder.* BILLROTH. (*Boston Med. and Surg. Reporter*, July 10.)

On June 3, 1874, a boy, æt. 12, was admitted to Prof. Billroth's clinic. He exhibited such symptoms as are known to be caused by a stone in the bladder. But on examination, a tumor about the size of a fist, evidently attached to the wall of the bladder, could be felt through the abdominal wall as well as per rectum. The beak of the sound, immediately on entering the bladder, was pushed forward and slid over an uneven tumor before reaching the back of the bladder. Billroth decided upon extirpating this growth, and on June 15, 1874, did so. First, the perineal section was performed; the diagnosis of a tumor springing from the posterior wall of the bladder was confirmed, but at the same time it was found impossible to remove the neoplasma by the perineal route. Therefore the bladder was opened a second time by a transverse incision in the supra-pubic region ; the bulk of the tumor was torn off by the finger and the pedicle carefully cut out, whereby the peritoneum fortunately was not injured. A drainage-tube was drawn through the bladder and kept there for five days until the wounds were granulating well. No severe reaction supervened, and on July 18, the boy was discharged. The tumor showed the consistence and appearance of a soft fibrous tumor, but under the microscope it exhibited a mixed structure of myoma and sarcoma.

11. *Transfusion.*

In a correspondence of the *Philadelphia Med. Times* (August 7, 1875,) from Strassbourg, Germany, the following experiment of Prof. Ponfick is given: A dog received into the jugular vein from the carotid of another animal, during forty-five seconds, twelve per mille of blood of the weight of the dog. During the reception of the blood there was moderate dyspnœa, extreme nausea, but no vomiting; the extremities seemed paralyzed. After an hour, bloody coloration of conjunctiva of both eyes ; some red coloration of urine forty-five hours after operation. During first day, dog seemed to be doing well, but suddenly collapsed at the end of twenty-eight hours; respiration became superficial, weak and infrequent, pupils widely dilated, occasional convulsions, and the dog died at the end of eighty hours after operation. Postmortem examination showed *severe renal disease.* Other experiments have resulted similarly whether the blood of the other animal has been transfused with or without defibrination. The conclusion drawn from all these experiments is, that the serum of one animal dissolves the blood-cells of other species, and that for transfusion into men, human blood only should be used.

12. *Statistics of Amputations* performed at the Glasgow Royal Infirmary during the twenty-five years ending 31st December, 1873. (*Glasgow Med. Journal*, April, 1875.)

There have been 1,412 amputations, of which 67.9 per cent. recovered, and 32.1 per cent. died. Of the 657 primary amputations, 36.5 per cent. died; of 172 secondary amputations, 51.7 per cent. died; and of 583 amputations for disease, 21.9 per cent. died.—*Monthly Abstract Med. Sciences*, July, 1875.

13. *The Antiseptic Treatment at the Surgical Clinic in Halle, Germany.*

At a recent visit of Prof. Lister in Halle, Prof. Volkmann, who is one of the strongest believers in Lister's antiseptic treatment, gave the following interesting statistics: 44 complicated fractures treated conservatively, all recovered; 67 amputations, with death in 6 cases only; 16 osteotomies, none died; 28 excisions of joints, 7 died.

